# Supplementary material for: WordCommentsAnalyzer: A windows software tool for qualitative research
Source: F1000Res. 2018 Sep 4;7:536. Originally published 2018 May 3. [Version 2] doi: 10.12688/f1000research.14819.2 (PMC6137411; doi:10.12688/f1000research.14819.2)
Supplement: Supplementary file 3 [file f1000research-7-17614-s0002.tgz › b2a79496-4ada-47c4-9682-a99ce867836d.zip!/Eight Iranian Health Journals/Journal of Health System Research _6.docx]

© Journal of Health System Research . This work is licensed under CC BY-NC 4.0 (https://creativecommons.org/licenses/by-nc/4.0/).

Volume & Issue: دوره 12، شماره 4، 1395

Title: کاربرد انبار داده در حوزه سلامت کشور ایران: چالش‌ها و راهکارها

http://hsr.mui.ac.ir/index.php/jhsr/article/view/2728

مقدمه: داده‏ها در نظام سلامت به صورت پراکنده و جزیره‏ای قرار دارند و دسترسی یکپارچه به آن‌ها برای گزارش‏گیری و تحلیل مشکل است. انبار داده می‌تواند در حل این مسایل کمک کننده باشد. بنابراین، مطالعه حاضر به شناسایی چالش‌ها و راهکارهای کاربرد انبار داده در حوزه سلامت کشور پرداخت. روش‌ها: این مطالعه به صورت مروری جامع و نقلی انجام شد. ابتدا واژه‌های کلیدی در حیطه انبار داده‌ها انتخاب گردید و به لحاظ گستردگی موضوعی انبار داده‌ها، دامنه جستجو به حوزه سلامت محدود شد. سپس با توجه به معیارهای مطالعه که اغلب استفاده از مطالعات کشورهای توسعه یافته بود، مقالات مرتبط با موضوع از پایگاه‌های Google Scholar، PubMed، Scopus، Web of Knowledge و Science Direct استخراج گردید. یافته‌ها: اغلب مؤسسات مراقبت سلامت با چالش‏های جمع‏آوری، سازماندهی و بازیابی داده‏های نامتجانس و حجیم برای تحقیقات، بهبود کیفیت، تحلیل برون‌دادها یا کشف بهترین عملکردها مواجه هستند. ‏راه‌حل بیشتر چالش‌های ذکر شده، انبار داده است. این تکنیک می‏تواند بسیاری از مشکلات حیطه مدیریت داده‏ها و بهره‏برداری از دانش داده‏ای نظام سلامت را مرتفع ‏نماید. نتیجه‌گیری: به کارگیري سیستم‌هایی مانند انبار داده براي پیشگیري از پیشرفت یا ظهور بیماري و سلامت جامعه، هدف اصلی نظام سلامت است. پژوهش حاضر گامی ابتدایی در راستای به کارگیری انبار داده ملی سلامت می‌باشد و بی‌تردید نیازمند نگرش ملی برای حل چالش‌ها و ارایه راهکارهای مناسب می‌باشد.

Title: بررسی نحوه‌ انسداد خطوط فاضلاب شهری در اثر تجمع رسوب‌های روغن و چربی و روش‌های مقابله با آن

http://hsr.mui.ac.ir/index.php/jhsr/article/view/2551

مقدمه: گرفتگی‌های ناشی از رسوب روغن و چربی در خطوط فاضلاب، بخش عمده‌ای از کل گرفتگی‌های خطوط فاضلاب را در دنیا تشکیل می‌دهد. به‌ عنوان مثال، در بریتانیا و آمریکا حدود 50 درصد از گرفتگی‌ها به ‌دلیل رسوب روغن و چربی است. روش‌های مختلفی برای حل این مشکل وجود دارد که هر کدام مزایا و معایب خود را دارند. هدف از انجام پژوهش حاضر، دستیابی به دستورالعمل‌های اجرایی برای کاهش مشکلات ناشی از ایجاد رسوب چربی و روغن در شبکه‌های جمع‌آوری فاضلاب شهری بود. روش‌ها: تمام مقالات مرتبط مورد استفاده در این پژوهش از پایگاه‌های اطلاعاتی Science Direct، Google Scholar و Free Patents Online جستجو شد و مورد بررسی قرار گرفت. در نهایت، حدود 50 مقاله و گزارش که با موضوع مرتبط بودند، استفاده‌ گردید. در این میان، تجربیات عملی نویسندگان و مشاوره با کارشناسان خبره نیز کمک شایانی برای دستیابی به نتایج مناسب داشت. یافته‌ها: بررسی منابع علمی به‌ همراه تجربیات عملی نویسندگان در حل معضلات مربوط به ایجاد رسوب چربی و روغن در شبکه‌‌ جمع‌آوری فاضلاب شهری به ‌ویژه در شهر مشهد و مشاوره با کارشناسان خبره در این مورد نشان داد که به ‌منظور پیشگیری از انسداد خطوط فاضلاب ناشی از تشکیل این رسوب‌ها، چهار روش وجود دارد که شامل «اجرای دستورالعمل مدیریت پسماند روغن و چربی و نصب دستگاه چربی‌گیر در آشپزخانه، طراحی مناسب خطوط فاضلاب جدید و اصلاح خطوط فاضلاب موجود و برنامه شستشوی پیشگیرانه‌ منظم» بود. نتیجه‌گیری: انسداد خطوط فاضلاب ناشی از تشکیل رسوب‌های چربی و روغن، یکی از مشکلات بزرگ صنعت آب و فاضلاب در دنیا و کشور است. راهکارهای مختلفی برای مقابله با این مشکل پیشنهاد شده است. استفاده‌ هم‌زمان از چند روش پیشنهادی در پژوهش حاضر برای دستیابی به بالاترین بهره‌وری و نیز کم‌ترین هزینه‌ ممکن، می‌تواند ضمن کاهش یا جلوگیری از وقوع مشکلات احتمالی، هزینه‌های مالی و زیست محیطی ناشی از آن را کاهش دهد.

Title: فراتحلیل تحقیقات انجام شده در مورد رابطه حمایت اجتماعی و کیفیت زندگی در سالمندان ایران

http://hsr.mui.ac.ir/index.php/jhsr/article/view/2252

مقدمه: هدف از انجام پژوهش حاضر، فرا تحلیل تحقیقات انجام شده درباره رابطه حمایت اجتماعی وکیفیت زندگی در سالمندان ایران بود. برای دستیابی به این هدف، دو سؤال در مورد اندازه اثر رابطه حمایت اجتماعی، کیفیت زندگی در سالمندان ایران و مقایسه میزان رابطه انواع حمایت اجتماعی باکیفیت زندگی سالمندان طرح شد. روش‌ها: به منظور پاسخگویی به سؤال‌های فراتحلیل، از مجموعه پژوهش‌های انجام شده درباره رابطه حمایت اجتماعی وکیفیت زندگی در سطح کشور در فاصله سال‌های 93-1388، 7 پژوهش که از لحاظ روش‌شناختی مورد قبول بود و شرایط ورود به فراتحلیل را داشت، برای انجام فراتحلیل انتخاب شد. این 7 پژوهش، حاوی 15 اندازه اثر در مورد رابطه حمایت اجتماعی و انواع آن باکیفیت زندگی در سالمندان بود. حجم نمونه مجموع این تحقیقات از 1852 نفر تشکیل شد. برای گردآوری اطلاعات از چک‌لیست فراتحلیل استفاده شد. یافته‌ها: بر اساس نتایج این فراتحلیل، اندازه اثر رابطه حمایت اجتماعی و کیفیت زندگی در سالمندان 33/0 بود (00001/0 > P). همچنین، نشان داد که در سالمندان اندازه اثر رابطه حمایت عاطفی و کیفیت زندگی 29/0 (00001/0 > P)، اندازه اثر رابطه حمایت ابزاری و کیفیت زندگی 18/0 (00001/0 > P) و اندازه اثر رابطه حمایت اطلاعاتی و کیفیت زندگی 20/0 (0001/0 > P) به دست آمد. نتیجه‌گیری: اندازه اثر رابطه حمایت اجتماعی و کیفیت زندگی در سالمندان ایران در کل و در مورد حمایت عاطفی، بر اساس معیار تفسیر Cohen در حد متوسط است. این امر اهمیت فراهم کردن زمینه تقویت حمایت اجتماعی برای بهبود کیفیت زندگی سالمندان ایرانی را خاطر نشان می‌سازد.

Title: بررسی رابطه بین حمایت اجتماعی با رفتارهای تغذیه‌ای در دختران دانش‌آموز دارای اضافه وزن و مبتلا به چاقی مدارس راهنمایی شهر شاهین‌شهر

http://hsr.mui.ac.ir/index.php/jhsr/article/view/2256

مقدمه: شیوع اضافه وزن و چاقی در کودکان و نوجوانان سراسر جهان به میزان قابل توجه و هشدار دهنده‌ای رسیده است. یکی از مهم‌ترین شاخص‌های روان‌شناختی مؤثر در تغییر رفتارهای تغذیه‌ای نوجوانان، حمایت اجتماعی معرفی شده است. بنابراین، هدف از انجام مطالعه حاضر، بررسی رابطه بین حمایت اجتماعی با رفتارهای تغذیه‌ای در نوجوانان دارای اضافه وزن و چاق شهر شاهین‌شهر بود. روش‌ها: این مطالعه مقطعی بر روی 172 دانش‌آموز دختر دارای اضافه وزن و مبتلا به چاقی شهر شاهین‌شهر، در سال 1392 انجام شد. پس از نمونه‌گیری تصادفی و ارزیابی شاخص‌های تن‌سنجی، پرسش‌نامه مربوط به حمایت اجتماعی و تکرر مصرف وعده‌های غذایی توسط دانش‌آموزان تکمیل گردید. همچنین، از دانش‌آموزان یادآمد 24 ساعته غذایی گرفته شد. یافته‌ها: میانگین سنی و شاخص توده بدنی (Body mass index یا BMI) دانش‌آموزان شرکت کننده به ترتیب 6/0 ± 4/13 سال و 6/3 ± 2/28 کیلوگرم بر مترمربع بود. بین میزان BMI با مصرف صبحانه و میان وعده‌های صبح و عصر ارتباط معکوس و معنی‌داری مشاهده شد (001/0 > P). همچنین، حمایت اجتماعی با مصرف صبحانه و میان وعده‌های صبح و عصر ارتباط مثبت و معنی‌داری داشت (001/0 > P)، اما رابطه بین مصرف میوه و سبزی و تنقلات کم‌ارزش با حمایت اجتماعی معنی‌دار نبود. نتیجه‌گیری: حمایت اجتماعی به طور مؤثری مصرف صبحانه در دانش‌آموزان چاق را پیش‌بینی نمود. به نظر می‌رسد که برای درک بهتر رابطه بین حمایت اجتماعی با رفتارهای تغذیه‌ای، در آینده نیاز به مطالعات بیشتری در این زمینه می‌باشد.

Title: تعیین میزان انتشار گازهای مونواکسید کربن و دی‌اکسید کربن حاصل از سوخت مازوت در کارخانه سیمان دورود لرستان و شبیه سازی الگوی پراکنش گاز مونواکسید کربن

http://hsr.mui.ac.ir/index.php/jhsr/article/view/2294

مقدمه: کارخانه سیمان دورود به عنوان یکی از منابع مهم گازهای آلاینده، در استان لرستان واقع شده است. در این کارخانه در فصل زمستان به دلیل کاهش فشار گاز طبیعی، از مازوت به عنوان سوخت کوره استفاده می‌گردد. هدف از انجام مطالعه حاضر، بررسی میزان انتشار گاز دی‌اکسید کربن (2CO) حاصل از کاربرد سوخت مازوت از دودکش کارخانه سیمان دورود لرستان در فصل زمستان و شبیه‌سازی انتشار گاز مونوکسید کربن (CO) در شرایط مختلف جوی در جهت پایین دست باد توسط مدل SCREEN3 بود. روش‌ها: این مطالعه به روش توصیفی- تحلیلی انجام گرفت. غلظت‌های حجمی گازهای مونوکسید کربن و دی‌اکسید کربن اندازه‌گیری شده توسط Testo XL350 به مقدار نشر جرمی تبدیل گردید و با استاندارد خروجی از الکتروفیلتر و کوره مقایسه شد. مقادیر نشر جرمی گاز مونوکسید کربن در سه دودکش کارخانه سیمان در کلاس‌های مختلف پایداری که احتمال رخداد آن در فصل زمستان وجود داشت، با استفاده از مدل نرم‌افزاری SCREEN3 شبیه‌سازی شد. یافته‌ها: میزان نشر جرمی به دست آمده از گاز دی‌اکسید کربن بالاتر از استاندارد خروجی الکتروفیلتر و پیش گرمکن گزارش گردید. یافته‌های حاصل از مدل‌سازی گاز مونوکسید کربن در جهت پایین دست باد غالب نشان داد که حداکثر مقدار مشاهده شده در کلیه شرایط اتمسفری در فاصله 4924 متری، برابر با 81/778 میکروگرم بر مترمکعب در مسیر باد غالب از سمت جنوب غربی بود. نتیجه‌گیری: با توجه به این که بیشترین غلظت پیش‌بینی شده گاز مونوکسید کربن در کلیه شرایط اتمسفری در فاصله 4924 متری دودکش به دست آمد؛ می‌توان گفت که این نقطه به عنوان حساس‌ترین وآسیب‌پذیرترین نقطه در اطراف کارخانه سیمان از نظر انتشار این گاز می‌باشد. به منظور کنترل و کاهش غلظت خروجی گاز دی‌اکسید کربن، به اقدامات بهینه‌تری نیاز است.

Title: ارزيابي فرهنگ ايمني و تعيين متغير‌هاي فردي و شغلي پيش‌بيني كننده آن در صنايع استان قم

http://hsr.mui.ac.ir/index.php/jhsr/article/view/2426

مقدمه: فرهنگ ايمني شاخصي از عملكرد ايمني سازمان‌ها بوده و نقش اساسي در شكل‌گيري رفتار ايمني كاركنان دارد. بنابراين، ارزيابي فرهنگ ايمني و شناسايي متغير‌هاي مرتبط با آن مي‌تواند نقش مهمي در كاهش حوادث محيط كار داشته باشد. مطالعه حاضر با هدف ارزيابي فرهنگ ايمني و تعيین متغير‌هاي فردي و شغلي مرتبط با آن در كاركنان صنايع مختلف استان قم انجام شد. روش‌ها: مطالعه حاضر، توصیفی- تحلیلی به صورت مقطعی روي 289 نفر از كاركنان صنايع استان قم انجام شد. ابزار گرد‌آوري اطلاعات شامل پرسش‌نامه اطلاعات فردي- شغلي و پرسش‌نامه فرهنگ ايمني بود. آناليز داده‌ها در نرم‌افزار SPSS و با استفاده از آزمون‌هاي ضریب همبستگی Pearson، آزمون تي مستقل، آنالیز واریانس يك‌طرفه و رگرسيون خطي چندگانه انجام شد.   يافته‌ها: ميانگين امتياز فرهنگ ايمني برابر با 73/0 ± 30/3 بود. بيشترين امتياز در بعد مشاركت و همكاري (45/0 ± 53/3) و كمترين در بعد محيط و اولويت ايمني (86/0 ± 06/3) بود. اندازه صنعت، سابقه كار، مصرف دخانيات و ساعات كار روزانه داراي ارتباط معني‌دار با فرهنگ ايمني بود (01/0 > P) و در کل 9/19 درصد از تغييرات فرهنگ ايمني را تبيين مي‌كند. نتيجه‌گيري: با توجه به ارتباط متغير‌هاي مورد مطالعه با فرهنگ ايمني و از طرفي ارتباط فرهنگ ايمني با عملكرد ايمني و حوادث، اين متغير‌ها از عواملي هستند كه به طور مستقيم يا غير مستقيم بر روي بروز حوادث اثر دارد. بنابراين، نياز است كه نقش اين متغير‌ها در تدوين برنامه‌هاي كنترل حوادث و تصميم‌گيري جهت ارتقای ايمني در نظر گرفته شود.

Title: بررسی کارایی جذب پیروکاتیکول از محلول‌های آبی با استفاده از پامیس (طبیعی و اصلاح شده با اسید کلریدریک)

http://hsr.mui.ac.ir/index.php/jhsr/article/view/2450

مقدمه: پیروکاتیکول در محیط‌های آبی برای موجودات زنده و انسان خطرناک می‌باشد. بنابراین، حذف آن از فاضلاب‏ها و جلوگیری از ورود آن به محیط حایز اهمیت است. هدف از انجام مطالعه حاضر، بررسی کارایی جذب پیروکاتیکول از محلول‏های آبی با استفاده از پامیس (طبیعی و اصلاح شده با اسید کلریدریک) بود. روش‌ها: در این مطالعه تجربی، از پامیس با اندازه مش 40-20 به عنوان جاذب به صورت ناپیوسته در ارلن‌های 250 میلی‌لیتری استفاده شد. اثر شاخص‌های مؤثر در جذب مانند pH، زمان تماس، دوز جاذب و غلظت اولیه پیروکاتیکول مورد بررسی قرار گرفت. داده‌ها با ایزوترم Langmuir و Freundlich و سینتیک شبه درجه اول و شبه درجه دوم بررسی گردید. یافته‌ها: در غلظت اولیه 10 میلی‌گرم بر لیتر از پیروکاتیکول، حداکثر میزان جذب برای پامیس طبیعی بیش از 90 درصد و برای پامیس اصلاح شده با اسید کلریدریک بیش از 98 درصد به دست آمد. با افزایش زمان تماس و دوز جاذب در پامیس طبیعی و اصلاح شده، جذب افزایش یافت و در زمان 20 دقیقه به تعادل رسید. جذب به وسیله پامیس طبیعی و اصلاح شده با مدل ایزوترم Langmuir (برای پامیس طبیعی 974/0 = 2R و برای پامیس اصلاح شده 998/0 = 2R) همخوانی بیشتری را نشان داد. جذب با مدل سینتیکی شبه درجه دوم (برای پامیس طبیعی 9977/0 = 2R و برای پامیس اصلاح شده 9788/0 = 2R) مطابقت بیشتری داشت. نتیجه‌گیری: با توجه به این که پامیس ارزان و در دسترس است و در معادن ایران به وفور یافت می‌شود، می‌تواند گزینه مناسبی جهت حذف پیروکاتیکول از فاضلاب باشد. همچنین، اصلاح پیروکاتیکول به وسیله اسید کلریدریک، روش مناسبی برای افزایش جذب پیروکاتیکول با استفاده از پامیس است.

Title: بررسی ارتباط بین شیوع اختلالات اسکلتی- عضلانی و رضایت شغلی در رانندگان آژانس استان لرستان

http://hsr.mui.ac.ir/index.php/jhsr/article/view/2483

مقدمه: رانندگان تاکسی به دلیل ارتعاشات ناشی از ماشین، نشستن طولانی مدت، وضعیت بدنی نامناسب، خم شدن و چرخیدن مکرر حین انجام کار، در درازمدت در معرض خطر بروز اختلالات اسکلتی- عضلانی قرار دارند. بنابراین، مطالعه حاضر با هدف بررسی ارتباط بین شیوع اختلالات اسکلتی- عضلانی و رضایت شغلی در رانندگان آژانس استان لرستان انجام شد. روش‌ها: اين مطالعه مقطعی از نوع توصيفي- تحليلي بود كه بر روی 100 نفر از رانندگان آژانس استان لرستان صورت گرفت. جهت جمع‌آوری اطلاعات، از دو ابزار شامل پرسش‌نامه اسکلتی- عضلانی Nordic (Nordic Musculoskeletal Questionnaire یا NMQ) و پرسش‌نامه رضایت شغلی استفاده گردید. داده‌ها با استفاده از آزمون Independent t، همبستگی Pearson، همبستگی Spearman و آنالیز کوواریانس مورد تجزیه و تحلیل قرار گرفت. یافته‌ها: ضریب همبستگی Pearson نشان داد که بین نمره اختلالات اسکلتی- عضلاتی و نمره کلی رضایت شغلی رابطه معنی‌داری وجود نداشت (136/0 = r، 186/0 = P). همچنین، بین نمره اختلالات اسکلتی- عضلاتی با سن (54/0 = r، 001/0 > P) و سابقه کار (58/0 = r، 001/0 > P) ارتباط مستقیمی مشاهده شد. بیشترین میزان شیوع اختلالات اسکلتی- عضلانی در یک سال گذشته به ترتیب مربوط به نواحی کمر (96 درصد)، گردن (87 درصد)، زانو (84 درصد)، مچ دست (83 درصد) و شانه (81 درصد) بود. نتیجه‌گیری: بر اساس نتایج مطالعه حاضر، میزان بروز اختلالات اسکلتی- عضلانی در رانندگان بالا و رضایت افراد متوسط می‌باشد. از این‌رو، پیشنهاد می‌گردد که به منظور کاهش بروز اختلالات اسکلتی- عضلانی، از استراحت و نرمش‌های مناسب طی انجام وظیفه و طراحی صندلی ارگونومی بر اساس ابعاد آنتروپومتریک استفاده شود.

Title: مدیریت لجن اسیدی تولیدی در شهرک‌های صنعتی استان اصفهان

http://hsr.mui.ac.ir/index.php/jhsr/article/view/2504

مقدمه: مدیریت نامناسب پسماندهای صنعتی مشکلات فراوانی را برای جوامع بشری به وجود آورده است. مطالعه حاضر با هدف بررسی مدیریت لجن اسیدی تولید شده در شهرک‌های صنعتی استان اصفهان در سال 1393 انجام شد. روش‌ها: این تحقیق از نوع توصیفی- مقطعی بود. 180 واحد صنعتی در پنج شهرک صنعتی شامل اشترجان، مبارکه، رازی، جی و مورچه‌خورت مورد بازدید قرار گرفت و وضعیت صنایع با استفاده از پرسش‌نامه بررسی گردید. مقدار لجن اسیدی برای سایر شهرک‌های صنعتی با استفاده از میزان به دست آمده محاسبه شد. یافته‌ها: لجن اسیدی در 8 زیرگروه مختلف صنعتی بررسی شد. زیرگروه پارافین و حلال‌های آلی با 3092 تن در هکتار در سال، بیشترین و زیرگروه تصفیه روغن با 623 تن در هکتار در سال، کمترین میزان لجن اسیدی تولید شده را داشتند. شهرک رازی (2700 تن در سال) و اوره نطنز (62 تن در سال) به ترتیب بیشترین و کمترین میزان لجن تولیدی در زیرگروه پارافین و حلال‌های آلی را به خود اختصاص دادند. بیشترین و کمترین میزان تولید لجن در زیرگروه تصفیه روغن نیز متعلق به مورچه‌خورت (746 تن در سال) و علویجه (39 تن در سال) بود. نتیجه‌گیری: لجن اسیدی تولید شده از جمله مواد زاید خطرناک محسوب می‌گردد و به منظور کاهش اثرات نامطلوب آن در محیط، روش‌های مدیریتی پیشنهاد شده باعث کاهش حجم قابل ملاحظه‌ای در تولید لجن می‌شود.

Title: بررسی ضریب جذب صوتی نسبت‌های مختلف نانو کامپوزیت پلی استایرن/ نانو خاک رس در فرکانس‌های مختلف

http://hsr.mui.ac.ir/index.php/jhsr/article/view/2508

مقدمه: یکی از روش‌های کنترل صدا استفاده از مواد جاذب صدا (به خصوص فوم‌های پلیمری) می‌باشد. در سال‌های اخیر نانوکامپوزیت‌های پلیمری به دلیل بهبود خواص پلیمرها مورد توجه قرار گرفته است. مقاله حاضر، به بررسی تأثیر نانوخاک‌های رس بر روی ضریب جذب صوتی مواد پلیمری پرداخت. روش‌ها: با استفاده از تکنیک پلیمریزاسیون درجا نانوکامپوزیت پلی‌استایرن/ خاک رس سنتز شد. برای مقایسه ضریب جذب پلیمر استایرن نیز سنتز گردید. مواد سنتز شده با استفاده از روش قالب‌گیری تراکمی به فوم تبدیل شد. ضریب جذب صوتی فوم‌ها با استفاده از لوله امپدانس مشخص گردید. یافته‌ها: ضریب جذب پلیمر استایرن دارای پیک در محدوده فرکانسی 2000 تا 4000 هرتز می‌باشد. بیشترین میزان ضریب جذب در فرکانس 2500 هرتز به میزان 75/0 است. 5/0 درصد خاک رس باعث شد که بیشترین میزان ضریب جذب در فرکانس 2500 هرتز به میزان 89/0 رسید. در 3 درصد خاک رس بیشترین ضریب جذب در فرکانس 3150 هرتز به میزان 9/0 بود و در 7 درصد خاک رس نیز بیشترین ضریب جذب در فرکانس 2000 هرتز به میزان 54/0 به دست آمد. نتیجه‌گیری: با اضافه شدن 5/0 درصد خاک رس به پلیمر، فقط ارتفاع پیک فرکانس افزایش یافت. در 3 درصد خاک رس علاوه بر افزایش میزان ضریب جذب، مکان محدوده پیک فرکانسی جابه‌جا شد و در 7 درصد خاک رس نیز با تغییر محدوده پیک فرکانسی، افزایش میزان ضریب جذب مشاهده گردید.

Title: بررسی آگاهی، نگرش و عملکرد کارکنان بیمارستان های دانشگاه علوم پزشکی تهران در مورد مدیریت دفع پسماندهای بیمارستانی در سال 1393

http://hsr.mui.ac.ir/index.php/jhsr/article/view/2615

مقدمه: پسماندهاي بیمارستانی برای محیط زیست بسیار خطرناک می‌باشند. هدف از انجام مطالعه حاضر، تعیین آگاهی، نگرش و عملکرد کارکنان بیمارستان‌های تحت پوشش دانشگاه علوم پزشکی تهران در مورد مدیریت پسماندهای بیمارستانی بود. روش‌ها: این مطالعه توصیفی- مقطعی در سال 1393 در بیمارستان‌های تحت پوشش دانشگاه علوم پزشکی تهران انجام شد. ابزار جمع‌آوری اطلاعات، پرسش‌نامه‌ای سه قسمتی در خصوص مدیریت پسماند بیمارستانی بود. داده‌ها با استفاده از آزمون‌های Kruskal-Wallis و Mann-Whitney و همبستگی‌های Spearman و V کرامر (Cramer's V) مورد تجزیه و تحلیل قرار گرفت. یافته‌ها: بر اساس یافته‌ها، وضعیت آگاهی و نگرش در حد کم و عملکرد در حد متوسط بود. همچنین، نتایج آزمون Kruskal-Wallis نشان داد که بین آگاهی کارکنان بیمارستان‌ها در گروه‌های شغلی و تحصیلی با سابقه گذراندن دوره بهداشتی تفاوت معنی‌داری وجود نداشت. نتیجه‌گیری: با توجه به آگاهی و نگرش کم جامعه مورد بررسی در رابطه با مدیریت پسماندهای بیمارستانی، راه‌های افزایش آگاهی و بهبود نگرش و عملکرد کارکنان از طریق کلاس‌های آموزشی، پیشنهاد می‌گردد.

Title: بررسی رفتارهای مرتبط با ایمنی غذا در زنان خانه دار شهرستان کوهپایه (استان اصفهان) در سال1394

http://hsr.mui.ac.ir/index.php/jhsr/article/view/2643

مقدمه: با توجه به این که رعایت رفتارهای مرتبط با ایمنی غذا در افراد، می‌تواند سبب حفظ سلامت گردد، مطالعه حاضر با هدف تعیین رفتارهای مرتبط با رعایت ایمنی غذا در زنان خانه‌دار شهر کوهپایه (استان اصفهان) انجام شد. روش‌ها: این مطالعه به صورت مقطعی بین 150 زن خانه‌دار شهرستان کوهپایه که به روش نمونه‌گیری تصادفی منظم انتخاب شده بودند، انجام گرفت. ابزار جمع‌آوری داده‌ها، پرسش‌نامه‌ای چندبخشی حاوی اطلاعات دموگرافیک و رفتارهای مرتبط با ایمنی غذا (5 کلید برای غذایی سالم‌تر و ایمن‌تر، سازمان جهانی بهداشت) بود که توسط مشارکت کنندگان تکمیل گردید. در نهایت، داده‌ها با استفاده از آزمون‌های آماری در نرم‌افزار SPSS مورد تجزیه و تحلیل قرار گرفت. یافته‌ها: میانگین سنی شرکت کنندگان، 41/12 ± 03/41 سال بود. میانگین هر کلید شامل رعایت نظافت، جداسازی مواد خام و پخته، پخت کامل مواد، نگهداری در دمای سالم و آب و مواد سالم به ترتیب 97/2 ± 95/47، 50/1 ± 89/13، 89/1 ± 21/13، 99/1 ± 83/24 و 93/1 ± 12/17 به دست آمد. بین موارد مرتبط با ایمنی غذا، تنها رعایت نظافت و آب و مواد غذایی سالم با تحصیلات زنان ارتباط معنی‌داری را نشان داد. نتیجه‌گیری: با توجه به نتایج حاصل شده، رعایت ایمنی غذا در زنان خانه‌دار شهرستان کوهپایه در سطح مطلوبی قرار دارد. با این وجود، آموزش درست در خصوص برخی شاخص‌ها مانند شستن دست قبل از غذا و استفاده اصولی و صحیح از پارچه‌های تنظیف، می‌تواند مفید واقع شود.

Title: بررسي ارتباط وضعیت سلامت عمومی و کیفیت خواب کارگران با آمادگی فردی در مدیریت بحران

http://hsr.mui.ac.ir/index.php/jhsr/article/view/2702

مقدمه: بحران، يك واقعه پيش‌بيني نشده است که می‌تواند خسارات مالي و جاني در بر داشته باشد. يكي از اصلي‌ترين اقدامات در مديريت بحران، ميزان آمادگي فردي براي مقابله با بحران است. هدف اصلي از انجام مطالعه حاضر، بررسی ارتباط بین آمادگی فردی در مدیریت بحران با سلامت عمومی و کیفیت خواب بود. روش‌ها: این پژوهش یک مطالعه مقطعی، توصیفی- تحلیلی بود که در سال 1394 انجام شد. جامعه آماری، 310 نفر از پرسنل شرکت معدنی و صنعتی گل گهر بودند. از آمار حوادث در شش‌ماه اول سال 1394 به عنوان متغیری برای بررسی آمادگی فردی در مدیریت بحران استفاده گردید. ابزار مورد استفاده برای بررسی وضعیت سلامت عمومی و کیفیت خواب کارگران، پرسش‌نامه‌های وضعیت سلامت عمومی و شاخص کیفیت خواب Pittsburgh بود. تجزیه و تحلیل داده‌ها با نرم‌افزار SPSS انجام شد. یافته‌ها: میانگین سنی کارگران 4 ± 2/32 سال و میانگین سابقه کار افراد 1/4 ± 6/7 سال بود. میانگین شاخص توده بدنی در بین کارگران شرکت 72/2 ± 2/25 کیلوگرم بر متر مربع به دست آمد. شيوع موارد مشكوك به اختلالات رواني با استفاده از نمره برش 23 در كل و 6 در خرده مقياس، نشان داد كه 3/32 درصد از كارگران، مشكوك به اختلال رواني بودند. همچنین، 7/39 درصد از كارگران كيفيت خواب مطلوب و 3/60 درصد از آن‌ها كيفيت خواب نامطلوب داشتند. نتیجه‌گیری: افزایش سلامت عمومی، باعث بالا رفتن آمادگی فردی در مدیریت بحران و در نتیجه كاهش حوادث شده است، ولی ارتباط معنی‌داری بین کیفیت خواب و حوادث نشان نداد.

Title: اثر خرفه (Portulaca Oleracea) بر پروفایل چربی خون، قند خون و سطح CRP در مردان مبتلا به سندرم متابولیک: کارآزمایی بالینی تصادفی دو سویه کور

http://hsr.mui.ac.ir/index.php/jhsr/article/view/2765

مقدمه: عواملی همچون چاقی، افزایش چربی خون، افزایش قند خون و افزایش دور کمر، خطر بروز بیماری‌های قلبی- عروقی را افزایش می‌دهند. مطالعات نشان داده‌اند که چربی امگا 3، وقوع بیماری‌های قلبی- عروقی را کاهش می‌دهد. خرفه نوعی گیاه علفی است که برگ‌های غنی از اسید لینولئیک دارد. هدف از انجام تحقیق حاضر، بررسی تأثیر عصاره خرفه بر پروفایل‌های چربی خون، قند خون و سطح CRP (C-reactive protein) بود. روش‌ها: این کارآزمایی باینی تصادفی دو سویه کور، بر روی 80 مرد مبتلا به سندرم متابولیک انجام شد. بیماران به دو گروه مداخله و شاهد تقسیم شدند. گروه مداخله روزانه دو کپسول خرفه و گروه شاهد دو کپسول دارونما دریافت کردند. برای بررسی اثر مداخله، از آزمون ANOVA استفاده گردید. یافته‌ها: بر اساس یافته‌ها، میزان قند خون، سطح CRP و LDL (Low-density lipoprotein) در گروه‌های مداخله و شاهد کاهش یافت، اما این کاهش معنی‌دار نبود. سطح تری‌گلیسرید در هر دو گروه افزایش نشان داد، اما افزایش معنی‌داری مشاهده نشد. سطح HDL (High-density lipoprotein) در گروه مداخله افزایش و در گروه شاهد کاهش پیدا کرد که تفاوت بین دو گروه معنی‌دار نبود. کلسترول و Non-HDL cholesterol در گروه مداخله کاهش و در گروه شاهد افزایش نشان داد که تفاوت معنی‌داری بین دو گروه گزارش نشد. نتیجه‌گیری: نتایج تحقیق حاضر نشان داد که مصرف کپسول عصاره خرفه، نتوانست میزان قند خون، پروفایل چربی خون و سطح CRP را در مردان مبتلا به سندرم متابولیک کاهش دهد.

Title: بررسی اثر مصرف عصاره‌ چای سبز و چای ترش بر استرس اکسیداتیو، فشار خون و نمایه‌ توده‌ بدنی مردان ورزشکار با رویکرد پیشگیری از بیماری‌های قلبی- عروقی

http://hsr.mui.ac.ir/index.php/jhsr/article/view/2811

مقدمه: امروزه یکی از مهم‌ترین عوامل خطر بروز بیماری‌های قلبی- عروقی، استرس اکسیداتیو می‌باشد. مطالعات مختلف ارتباطی قوی میان افزایش مصرف فلاونوئیدهای گیاهی و کاهش خطر بروز انواع بیماری‌های مزمن را گزارش کرده‌اند. هدف از انجام مطالعه حاضر، بررسی اثر مصرف عصاره‌ چای سبز و چای ترش بر استرس اکسیداتیو، فشار خون و نمایه‌ توده‌ بدنی (Body mass index یا BMI) مردان ورزشکار بود. روش‌ها: در این پژوهش، ۵۴ مرد ورزشکار که دارای معیارهای ورود به مطالعه بودند، انتخاب شدند. سپس شرکت ‌کنندگان با استفاده از روش بلوک‌های تصادفی سه‌تایی، در ۳ گروه قرار گرفتند. گروه‌های مداخله و دارونما به ترتیب کپسول‌های ۴۵۰ میلی‌گرمی عصاره‌ چای سبز، عصاره‌ چای ترش و مالتودکسترین را دریافت کردند. سطح سرمی مالون دی‌آلدهید (Malondialdehyde یا MDA)، فشار خون و BMI شرکت ‌کنندگان در ابتدا و انتهای مطالعه اندازه‌گیری گردید. یافته‌ها: مصرف عصاره‌ چای سبز و چای ترش پس از شش هفته، توانست سطح MDA سرم گروه مداخله را نسبت به گروه دارونما به ‌طور معنی‌داری کاهش دهد (008/0 = P). با این ‌وجود، تنها مصرف عصاره‌ چای ترش فشار خون سیستولیک را به طور معنی‌داری کاهش داد (001/0 > P). مصرف هر دو عصاره چای، تأثیر معنی‌داری بر فشار خون دیاستولیک، وزن و میزان BMI نداشت. نتیجه‌گیری: مصرف عصاره‌ چای سبز و چای ترش با کاهش استرس اکسیداتیو، می‌تواند خطر بیماری‌های قلبی- عروقی را در مردان ورزشکار کاهش دهد.

Title: كاربرد نظريه رفتار برنامه‌ریزی ‌شده در ترغيب زنان باردار شهرستان دنا به زايمان طبيعي

http://hsr.mui.ac.ir/index.php/jhsr/article/view/2814

مقدمه: در سال‌های اخیر، میزان سزارین در تمام کشورهای جهان اعم از کشورهای در حال توسعه و توسعه یافته افزایش یافته است. بسياري از موارد سزارين بدون وجود دلایل موجه پزشكي انجام می‌گیرد. مطالعه حاضر با هدف بررسي تأثير آموزش بر اساس نظريه رفتار برنامه‌ریزی ‌شده، بر افزایش ميزان استقبال زنان باردار نخست‌زاي شهرستان دنا از زايمان طبيعي انجام شد. روش‌ها: اين مطالعه از نوع پیش تجربي بود. نمونه‌ها از طريق سرشماري زنان باردار نخست‌زا كه در سه ماه سوم بارداري، در مراكز بهداشتي- درماني شهرستان دنا داراي پرونده سلامت بودند، انتخاب شدند. 52 نفر از زنان باردار بر اساس معيارهاي ورود، در مطالعه شركت نمودند و پرسش‌نامه پیش‌آزمون بر اساس نظريه رفتار برنامه‌ریزی ‌شده برای آنان تكميل گردید. پس از تجزیه‌ و تحلیل داده‌های پیش‌آزمون و مشخص شدن سازه‌هایی كه نيازمند تأكيد بيشتري بودند، مداخله آموزشي مبتنی بر نظريه انجام گرفت. يك ماه پس از انجام مداخله، پرسش‌نامه‌های پس‌آزمون تكميل شد. داده‌ها با استفاده از آزمون‌های Paired t و McNemar مورد تجزیه ‌و تحلیل قرار گرفت. یافته‌ها: یافته‌ها اختلاف معنی‌داری را در خصوص تغيير نگرش، كنترل رفتاري درك شده و هنجارهاي انتزاعي بعد از مداخله آموزشي نشان داد (05/0 > P)، اما تفاوت معنی‌داری در مورد قصد رفتار قبل و بعد از آموزش مشاهده نشد (05/0 < P). پس از مداخله، 3/17 درصد از زنان قصد زايمان طبيعي و 1/73 درصد قصد سزارين داشتند. در نهایت، 35 درصد از زنان زايمان طبيعي و 65 درصد زايمان سزارين انجام دادند. نتیجه‌گیری: هرچند نتایج مطالعه حاضر نشان داد که آموزش مبتنی بر تئوری رفتار برنامه‌ریزی شده، باعث بهبود تمام سازه‌های پیشگویی کننده قصد رقتار می‌گردد، اما افزایش معنی‌داری در قصد رفتار مشاهده نشده است. بنابراین، پیشنهاد می‌شود که مداخلات و برنامه‌های آموزشی، بر اساس این تئوری و با تلفيق تئوری‌ها و الگوهاي مختلف مرتبط با موضوع طراحی و اجرا گردد.

Title: بررسی اثر جایگزینی ساکارز با شیرین کننده طبیعی استویا بر ویژگی‌های حسی و فیزیکوشیمیایی نوشابه گازدار پرتقالی

http://hsr.mui.ac.ir/index.php/jhsr/article/view/2864

مقدمه: امروزه استفاده از شیرین کننده طبیعی استویا، در بسیاري از مواد غذايی رژيمی به عنوان جايگزين شکر مورد توجه قرار گرفته است تا ضمن کمک به کاهش روند رو به رشد چاقی، در بهبود شیب افزایشی بیماری دیابت در جامعه نیز مؤثر باشد. هدف از انجام تحقیق حاضر، بررسی اثر جایگزینی ساکارز با شیرین کننده طبیعی استویا بر ویژگی‌های حسی و فیزیکوشیمیایی نوشابه گازدار پرتقالی بود. روش‌ها: به منظور انتخاب بهترین شیرین کننده با کمترین پس طعم، سه نمونه شیرین کننده استویای وارداتی به روش کروماتوگرافی مایع با کارایی بالا مورد آزمون قرار گرفت و نمونه با محتوای ربودیوزید بالاتر برای استفاده در فرمولاسیون نوشابه گازدار پرتقالی انتخاب شد. نوشابه‌های گازدار پرتقالی با جایگزینی 30، 50 و 70 درصد ساکارز با شیرین کننده استویا در دماهای 4، 25 و 37 درجه سلسیوس به مدت 1، 2 و 3 ماه نگهداری شد. سپس، نمونه‌ها از نظر pH، بریکس، دانسیته، اسیدیته و ویژگی‌های حسی مورد آزمون قرار گرفت. یافته‌ها: جایگزینی شیرین کننده طبیعی استویا در نوشابه‌های گازدار موجب تغییرات معنی‌داری در pH، بریکس، دانسیته و اسیدیته نوشابه‌ها گردیده، ولی رایحه و رنگ دچار تغییر نشد. همچنین، نگهداری نوشابه‌ها در دما و زمان در نظر گرفته شده، موجب کاهش pH و افزایش بریکس شد. با افزایش شیرین کننده استویا در سطح جایگزینی 30 درصد، تغییر قابل توجهی در پذیرش کلی نوشابه‌ها حاصل نشد، اما در سطوح بالاتر جایگزینی، پذیرش کلی کاهش یافت. نتیجه‌گیری: در صورت جایگزینی ساکارز با شیرین کننده طبیعی استویا و تولید نوشابه سبک حاوی 30 درصد استویا و 70 درصد ساکارز می‌توان کالری نوشابه را کاهش داد که به مراتب محصول مناسب‌تری نسبت به انواع تجاری رایج می‌باشد.

Title: آینده سازمان انتقال خون اصفهان تا سال 2030 با استفاده از تکنیک Delphi

http://hsr.mui.ac.ir/index.php/jhsr/article/view/2707

مقدمه: عبارت «آینده‌نگاری استراتژیک» امروزه به طور گسترده‌ای برای توصیف فعالیت‌ها و فرایندهایی استفاده می‌شود که به تصمیم‌ گیرندگان جهت هدایت طرح‌های آینده سازمان کمک می‌کند. پژوهش حاضر با هدف تعیین آینده سازمان انتقال خون و روندهای مثبت و منفی آن تا سال 2030 انجام شد. روش‌ها: این پژوهش توصیفی از نوع پیمایشی بود و از تکنیک Delphi به منظور پیش‌بینی آینده سازمان انتقال خون استفاده گردید. انجام این تکنیک پس از انجام دور چهارم و دستیابی به اتفاق نظر مطلوب، پایان یافت. نمونه‌های مورد مطالعه را مدیران و کارشناسان سازمان انتقال خون استان اصفهان و سه استان حادثه‌خیز منتخب دیگر از جمله آذربایجان شرقی، بوشهر و کرمان که دارای حداقل 10 سال سابقه کاری بودند، تشکیل داد که به صورت هدفمند انتخاب شدند. با استفاده از روش Delphi، لیستی از روندهای مثبت و منفی سازمان انتقال خون استان اصفهان تا سال 2030 مشخص گردید. سپس با استفاده از نرم‌افزار SPSS، میانگین هر یک از روندها مورد بررسی قرار گرفت. یافته‌ها: بر اساس یافته‌ها، مهم‌ترین روندهای مثبت سازمان انتقال خون تا سال 2030 شامل افزایش در تنوع محصولات خونی سازمان، افزایش تجهیزات و استفاده از فن‌آوری‌های نوین و IT در فرایندها، مدیریت بهینه مصرف خون و فرآورده‌های آن و تخصیص اعتبارات بیشتر و افزایش بودجه سازمان بود. همچنین، از جمله مهم‌ترین روندهای منفی این سازمان می‌توان به عدم نگاه و توجه مناسب سیاست‌گذاران به سازمان به عنوان یک سازمان حیاتی، ترک سازمان توسط افراد با قابلیت علمی بالا با توجه به کمبود حقوق و مزایا و عدم توسعه منابع انسانی اشاره نمود. نتیجه‌گیری: سازمان انتقال خون نیازمند یک برنامه استراتژیک بلند مدت و جذب منابع انسانی کارامد و فرهنگ‌سازی مناسب برای توجه به فعالیت‌های حیاتی سازمان انتقال خون و نقش آن در توسعه پایدار همه جانبه کشور است.

Title: بررسی روایی و پایایی پرسش‌نامه سواد سلامت مادری و نتایج بارداری

http://hsr.mui.ac.ir/index.php/jhsr/article/view/2828

مقدمه: سواد سلامت در مادران باردار به مهارت تشخیص نشانه‌های خطر دوران بارداری، شیوه زندگی سالم و تغذیه مناسب در این دوران گفته می‌شود که بر نتیجه بارداری تأثیر‌گذار است. مطالعه حاضر با هدف بررسی پایایی و روایی پرسش‌نامه سواد سلامت مادران و نتیجه بارداری (Maternal Health Literacy and Pregnancy Outcome یا MHLAP) انجام گردید. روش‌ها: پرسش‌نامه MHLAP به فارسی ترجمه و سپس ترجمه برگردان شد و روایی صوری و روایی محتوایی کمی و کیفی آن در یک مطالعه توصیفی- مقطعی مورد بررسی قرار گرفت. این بررسی با بهره‌گیري از نظرات اعضای هیأت علمی باسابقه و صاحب‌نظر انجام شد. همچنین، نسخه فارسی پرسش‌نامه در یک نمونه 113 نفره که به صورت تصادفی از بین 5 مرکز بهداشت شهرستان مشهد انتخاب شده بودند، مورد آزمون قرار گرفت. پایایی ابزار با استفاده از روش بازآزمایی به فاصله دو هفته و همسانی درونی نیز با استفاده از ضریب Cronbach¢s alpha سنجیده شد. براي ارزیابی روایی سازه از تحلیل عاملی اکتشافی و تأییدی استفاده گردید. یافته‌ها: ضریب Cronbach¢s alpha در بخش سنجش سواد سلامت، 89/0 و برای زیرشاخه‌ها 87/0 و 66/0 و در بخش نتیجه بارداری، 67/0 و برای زیرشاخه‌ها 72/0 و 69/0 به دست آمد. نتایج بازآزمایی حاکی از ثبات پرسش‌نامه و زیرشاخه‌ها بود. چرخش واریماکس در قسمت سواد سلامت، دو عامل و در قسمت نتیجه بارداری نیز دو عامل را نشان داد که در مجموع، در قسمت اول 82/54 و در بخش دوم 09/51 درصد واریانس را تبیین نمود. در هر دو قسمت پرسش‌نامه، نتایج تحلیل عاملی تأییدی بر اساس مدل دو عاملی به دست آمده از تحلیل عامل اکتشافی، برازش مناسبی با داده‌های حاصل شده داشت. نتیجه‌گیری: بر اساس نتایج به دست آمده، پرسش‌نامه MHLAP ابزاری پایا و روا می‌باشد و می‌تواند برای سنجش سواد سلامت و بررسی نتایج بارداری در مادران باردار مورد استفاده قرار گیرد.

Title: برآورد نااریب نسبت جمعیت‌های پنهان در معرض بیماری‌های پرخطر

http://hsr.mui.ac.ir/index.php/jhsr/article/view/2858

مقدمه: ازآن‌جا که سلامت جامعه در اثر بروز بیماری‌های پرخطر با مخاطره همراه می‌باشد، همواره جمعیت‌های در معرض این بیماری‌ها به خصوص جمعیت‌های پنهان، مورد توجه پژوهشگران و سیاست‌گذاران بهداشت عمومی قرار دارند. روش‌های متداولی که برای نمونه‌گیری و محاسبه برآورد نسبت این جمعیت‌ها مورد استفاده محققان قرار می‌گیرد، اغلب منجر به کم یا بیش‌برآوردی این نسبت‌ها در جمعیت‌های مورد مطالعه می‌گردد. با وجود معرفی روش‌های نمونه‌گیری کارایی همچون روش نمونه‌گیری پاسخگو محور از بیش از دو دهه پیش تاکنون، اما به دلیل عدم آشنایی پژوهشگران این حوزه با نحوه محاسبه برآوردها در نمونه‌های به دست آمده از این روش، کمتر از آن‌ در برآورد نسبت جمعیت‌های پنهان استفاده می‌گردد. هدف از انجام مطالعه حاضر، معرفی برآوردهای نسبت‌های جمعیتی برای متغیرهای کیفی مانند ابتلا به بیماری، با استفاده از برآوردهای احتمال روابط درون و برون‌گروهی و اندازه شبکه اجتماعی پاسخگویان بود. روش‌ها: با فرض وجود روابط دوطرفه در جمعیت هدف و انجام نمونه‌گیری با جای‌گذاری، در این مطالعه برآورد نسبت‌های جمعیتی با استفاده از برآوردهای احتمال روابط درون و برون‌ گروهی و اندازه شبکه اجتماعی پاسخگویان محاسبه گردید. یافته‌ها: تئوری‌های موجود و شبیه‌سازی‌های رایانه‌ای نشان داد که برآوردهای معرفی شده برای نسبت جمعیت‌های پنهان، به طور مجانبی نااریب بودند و نرخ همگرایی بالایی داشتند. نتیجه‌گیری: عدم انتخاب صحیح روش نمونه‌گیری و همچنین، محاسبه برآورد نسبت جمعیت‌های پنهان که در معرض بیماری‌های پرخطر هستند و در سیاست‌گذاری‌های بهداشتی تأثیرگذار می‌باشند، نتایج قابل قبولی در رسیدن به اهداف این سیاست‌گذاری‌ها ارایه نخواهد داد.
